# Gynecomastia and Klinefelter Syndrome

**Published:** 2015-12-16

**Authors:** Carol J. Singer-Granick, Tom Reisler, Mark Granick

**Affiliations:** ^a^Division of Pediatric Endocrinology, Department of Pediatrics, New Jersey Medical School, Rutgers University, Newark; ^b^Division of Plastic and Reconstructive Surgery, Department of Surgery, New Jersey Medical School, Rutgers University, Newark

**Keywords:** gynecomastia, Klinefelter syndrome, psychological, surgery, screening

## DESCRIPTION

**Figure 1:**
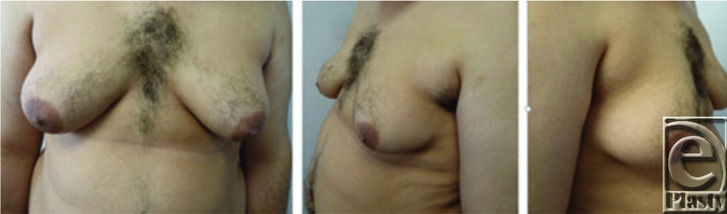
A preoperative photograph of our 22-year-old patient with Klinefelter syndrome demonstrating a grade III gynecomastia.

**Figure 2:**
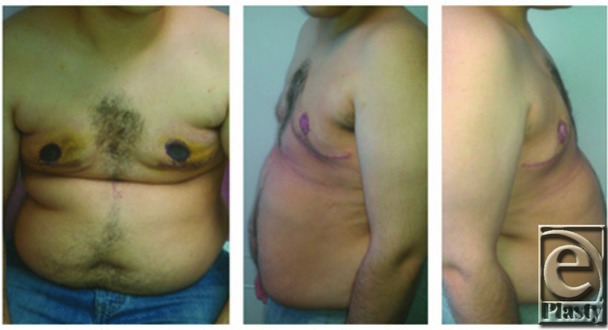
Postoperative photographs.

A 22-year-old man with known Klinefelter syndrome desired corrective surgery for gynecomastia. He had had marked gynecomastia with severe skin redundancy (Fig 1) for the past several years. The patient had suffered considerable psychological stress related to his condition. The gynecomastia was treated with simple mastectomies and nipple grafting (Fig 2).

## QUESTIONS

1. **What is the endocrinopathy in Klinefelter syndrome that leads to gynecomastia?**
2. **What are the psychiatric implications of gynecomastia?**
3. **What is the differential diagnosis of gynecomastia?**
4. **What techniques are available for male breast reduction?**

## DISCUSSION

Klinefelter syndrome is the most common sex chromosomal disorder in males and is caused by a chromosomal abnormality in which 2 or more X chromosomes are present along with a Y chromosome. The most common karyotype is 47XXY. Mosaic karyotypes, such as 46XY/47XXY, can also be seen but with less pronounced clinical features. Klinefelter syndrome occurs in 1:660 men and is the most common cause of chromosomal infertility in males.^1^ Klinefelter syndrome is usually diagnosed in puberty when the clinical features become most apparent. These include gynecomastia, eunuchoid body proportions, sparse facial and pubertal hair, and small firm testes. Gynecomastia results from a decreased testosterone to estradiol ratio. The seminiferous tubules of the testes hyalinize and fibrose, while adenomatous changes occur in the Leydig cells. This leads to impaired spermatogenesis and testosterone production. Adolescents have low testosterone and elevated gonadotropins (luteinizing hormone and follicle-stimulating hormone). The diagnosis is made by peripheral blood karyotype. The rare instances of mosaicism may require gonadal biopsy for diagnosis. It is important to know that patients with Klinefelter syndrome have an increased risk of breast cancer, as well as mediastinal and retroperitoneal germ cell tumors. Autoimmune disease and diabetes mellitus are more common. Patients with Klinefelter syndrome also have an increased incidence of neurodevelopmental issues and learning disabilities.^2^

Adult men with gynecomastia have psychological distress associated with the disorder,^2^ deriving from the significant impact on their lives during adolescence. Adolescents with gynecomastia are frequently affected emotionally and psychologically, regardless of graded severity of disease. Patients have reported embarrassment, humiliation, rejection, and teasing as a result of their breast development. In addition, there are increased feelings of loneliness, restlessness, and tension. There has been a higher association of depression, anxiety, adjustment disorders, low self-esteem, and suicidal ideation.^4^ It is essential to provide counseling, support, and treatment for all these patients.

Gynecomastia is frequently seen by plastic surgeons in its idiopathic form. However, it is critical for all surgeon who operate on these patients to understand that there is a lengthy and complex list of etiologies and numerous conditions associated with this clinical finding (Tables 1 and 2).^5^

Early intervention and treatment are necessary to improve the negative physical and emotional symptoms. Surgical options for gynecomastia vary due to the amounts of glandular, fibrous, adipose, and skin tissues involved. Liposuction has eliminated the need for skin resection in many patients with gynecomastia, especially adolescents. Fibrous and glandular enlargement can be managed with direct excision through areolar or remote incisions with adjunctive liposuction. However, skin resection is still recommended in patients with grade III gynecomastia^6^ who have significant ptosis. Procedures such as resection of a concentric circle of skin, pedicled relocation of the nipple with skin resection, or breast amputation with free nipple grafting are options. The treatment of gynecomastia requires an individualized approach.

Gynecomastia is a common clinical finding but has many uncommon etiologies. Klinefelter syndrome is just one of many conditions associated with gynecomastia. Since the diagnosis of Klinefelter syndrome often occurs in puberty, plastic surgeons may be the first physicians in a position to diagnose the disorder. A peripheral blood test for karyotyping and screening of endocrinopathies^5^ should be performed when additional physical findings suggest the diagnosis. The risk of concomitant breast cancer, germ cell tumors, diabetes, and autoimmune disease must also considered and investigated if indicated.

## Figures and Tables

**Table 1.** Conditions associated with gynecomastia

*Physiological*
Neonatal
Pubertal
Involutional
*Pathological*
Neoplasms
Testicular
Pituitary
Breast tumors
Adrenal
Liver
Human chorionic gonadotropin—ectopic production
Lymphoma/leukemia
Endocrinopathies
Hypogonadism
Syndrome: Klinefelter, Kallman's
Androgen insensitivity
Hermaphroditism
Enzymatic defects of testosterone synthesis
Testicular injury/regression
Hyperthyroidism
High aromatase
Adrenal hyperplasia
Corticotropin deficiency
Chronic illnesses
Liver disease
Renal disease
Malnutrition
Cystic fibrosis
AIDS
Ulcerative colitis
Medications

**Table 2:** Etiologies of gynecomastia

Idiopathic gynecomastia (no detectable abnormality)	25%
Pubertal gynecomastia	25%
Secondary to medication	10%–20%
Cirrhosis or malnutrition	8%
Primary hypogonadism	8%
Testicular tumors	3%
Secondary hypogonadism	2%
Hyperthyroidism	1.5%
Chronic renal disease	1%
